# Harnessing the value of TCTP in breast cancer treatment resistance: an opportunity for personalized therapy

**DOI:** 10.20517/cdr.2023.21

**Published:** 2023-07-13

**Authors:** Gianluca Santamaria, Mario Cioce, Antonia Rizzuto, Vito Michele Fazio, Giuseppe Viglietto, Maria Lucibello

**Affiliations:** ^1^Department of Experimental and Clinical Medicine, “Magna Graecia” University of Catanzaro, Catanzaro 88100, Italy.; ^2^Department of Medicine, Laboratory of Molecular Medicine and Biotechnology, University Campus Bio-Medico of Rome, Rome 00128, Italy.; ^3^Institute of Translational Pharmacology, National Research Council of Italy (CNR), Rome 00133, Italy.; ^4^Department of Medical and Surgical Sciences, “Magna Graecia” University of Catanzaro, Catanzaro 88100, Italy.; ^5^Department of Biomedical Sciences, Institute for Biomedical Research and Innovation, National Research Council of Italy (CNR), Catanzaro 88100, Italy.; ^#^These authors contributed equally.

**Keywords:** Breast cancer, metastasis, TCTP, stem cells, therapy resistance, biomarker

## Abstract

Early identification of breast cancer (BC) patients at a high risk of progression may aid in therapeutic and prognostic aims. This is especially true for metastatic disease, which is responsible for most cancer-related deaths. Growing evidence indicates that the translationally controlled tumor protein (TCTP) may be a clinically relevant marker for identifying poorly differentiated aggressive BC tumors. TCTP is an intriguing protein with pleiotropic functions, which is involved in multiple signaling pathways. TCTP may also be involved in stress response, cell growth and proliferation-related processes, underlying its potential role in the initiation of metastatic growth. Thus, TCTP marks specific cancer cell sub-populations with pronounced stress adaptation, stem-like and immune-evasive properties. Therefore, we have shown that *in vivo* phospho-TCTP levels correlate with the response of BC cells to anti-HER2 agents. In this review, we discuss the clinical relevance of TCTP for personalized therapy, specific TCTP-targeting strategies, and currently available therapeutic agents. We propose TCTP as an actionable clinically relevant target that could potentially improve patient outcomes.

## INTRODUCTION

Identification of crucial genes with therapeutic implications at an early stage of disease is a central challenge in tailoring therapeutic strategies. Estrogen receptor (ER) expression, progesterone receptor (PR) expression, and human epidermal growth factor receptor-2 (HER2) overexpression or amplification are well-established biomarkers that drive treatment decisions for patients with breast cancers (BC)^[1]^. Multi-parameter genomic assays, such as Oncotype DX and MammaPrint, are being used for patients with hormone receptor-positive (ER^+^/PgR^+^) and HER2^-^ early breast cancer who may benefit from adding chemotherapy to adjuvant endocrine therapy^[2,3]^. Specific gene signatures are still missing for non-ER^+^/HER^-^ clinical BC subgroups because, among those reported in the literature, none are in clinical use^[4]^.

Besides genomic studies, transcriptomic, proteomic and metabolomic analysis, or multi-omics data are all useful to discover biomarkers with an unprecedented level of complexity, as required for the current knowledge landscape of diseases. Further, post-translational modifications or subcellular localizations shape the level and the functional state of a protein, which cannot be detected by genomics-based approaches^[5]^. Last but not least, the relationship between protein and mRNA is very complex and highly influenced by cell types and cell states. Thus, transcript levels by themselves should not be deemed sufficient to predict protein levels^[6,7]^.

There are two current frontiers of cancer therapy, on one side, identifying clinically relevant alteration and, on the other side, doing this as early as possible during the progression of the disease. For example, despite advances in breast cancer detection and treatment, predicting which patients will develop overt metastatic disease remains a challenge. This is not simply a formidable technological challenge; it implies fundamental questions related to the degree of similarity between metastatic tissues and its primary source. However, it appears clear that prometastatic mutations may be represented very early in the primary tumor. For instance, Estrogen Receptor 1 (ESR1) mutation status, which has been associated with acquired resistance to endocrine therapy, is present not only in metastatic lesions but also in primary BC^[8]^.

After this general introduction, we intend to make the case here for translationally controlled tumor protein (TCTP), whose complex biology and link to clinical parameters may represent a good and practical example of the mentioned concepts.

We start from clinical observations highlighting the promising role of TCTP as a clinically relevant prognostic and predictive biomarker for identifying BC tumors at a high risk of progression early on. We also discuss signaling pathways that may impinge on stemness, drug resistance and poor response to immunotherapy. Finally, we explore strategies for targeting TCTP and the consequences of affecting TCTP expression in aggressive cancer cells.

## CLINICAL RELEVANCE OF TCTP IN CANCER

**Figure 1:**
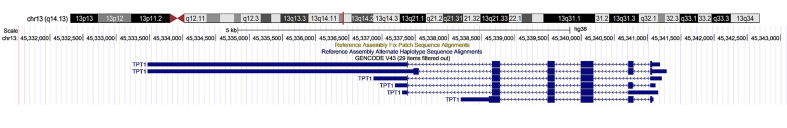
Genomic organization of TPT1. The UCSC genome browser was used to display the location and genomic organization of TPT1 on chromosome 13q14. Scheme of the TPT1 major types of alternative splicing is displayed (http://genome.ucsc.edu). UCSC Genome Browser on Human (GRCh38/hg38). TPT1: Tumor protein, translationally-controlled 1.

TCTP, also known as histamine-releasing factor (HRF), fortilin, P23, is encoded by the *TPT1* gene located on chromosome 13q12→q14^[9]^. The gene produces two TCTP mRNA isoforms which are different in the length of their 3’-UTR [Figure 1]. In this present work, we will refer, for convenience, to the shorter isoform, which is the most generally expressed in normal and cancer tissues^[10]^.

**Figure 2:**
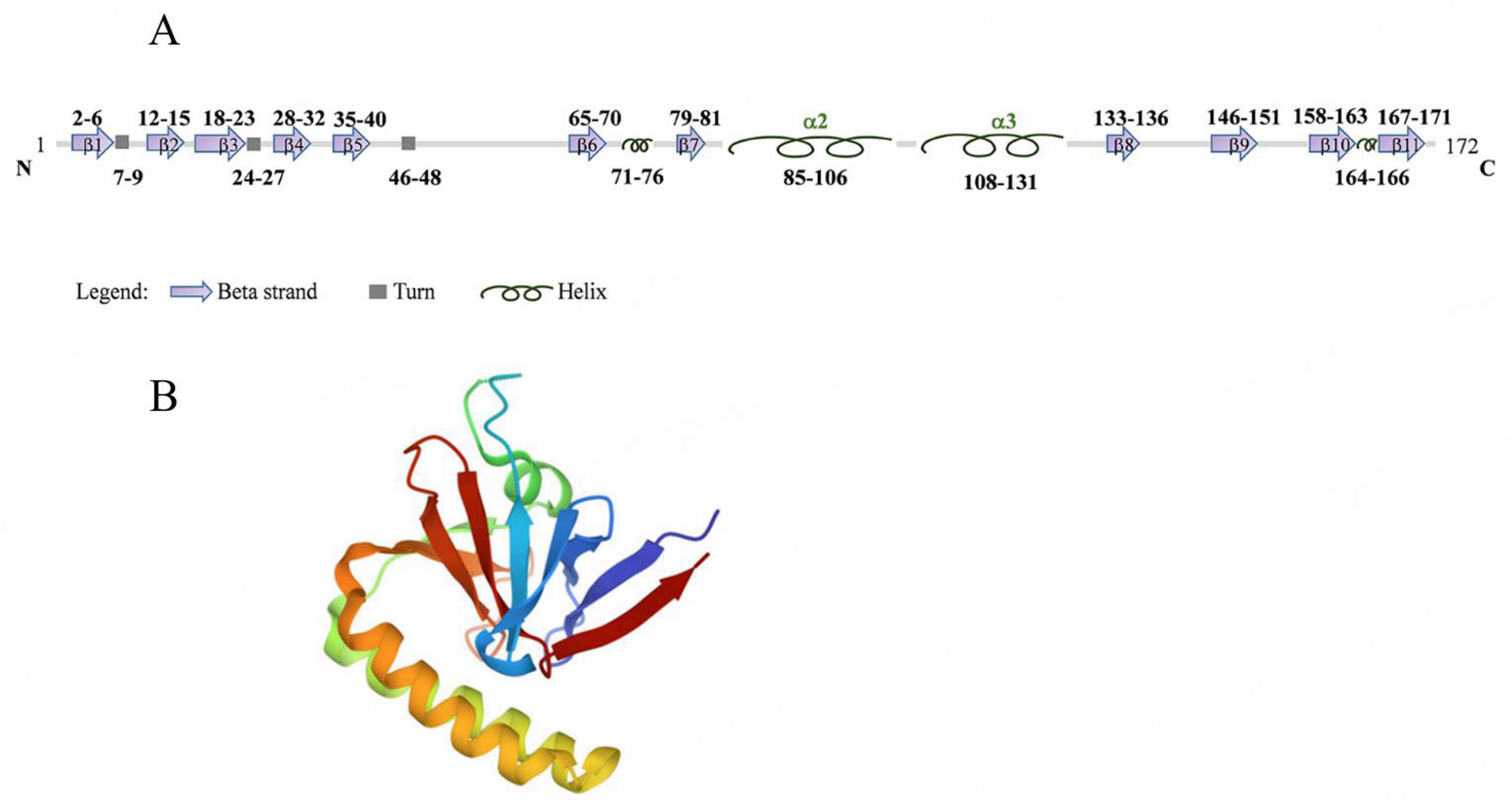
The peculiar structure of TCTP. (A) A cartoon representation of the secondary structure of human TCTP. Reference sequence from UniProt P13693^[11]^. β-strands, α-helices and turns elements are indicated in the legend. The N-terminal region includes a flexible loop extending from β5 to β6 strands. It contains a highly conserved signature and the Ser46 and Ser64 residues which are phosphorylated by the polo-like kinase Plk1, a crucial player in mitosis. A second conserved signature is found in the C-terminal region; (B) The crystal structure of the human TCTP (PDB Code 1YZ1^[12]^) discloses the α-helical hairpin (formed by Helix H2 and Helix H3), whose structure is similar to the H5-H6 helices of BCL-2 family proteins, and the β-stranded domain that shows a structural analogy with the guanine nucleotide exchange factors (GEF) Mss4/Dss4 protein families^[13]^, suggesting a similar role for TCTP as GEF for Ras homolog enriched in brain (Rheb) in the mTORC1 pathway^[14]^. The flexible loop is not detectable in the crystal structure. TCTP: Translationally controlled tumor protein.

The gene encodes the protein TCTP, which is highly conserved throughout evolution and without any sequence homology to other proteins [Figure 2].

### TCTP as a prognostic marker

Emerging clinical evidence has shown that the TCTP protein expression was dysregulated in many diseases^[15]^, including various hematological^[16,17]^ and solid tumors [Table 1]. Further, a high TCTP status was positively correlated with the pathological grade and markedly associated with shorter overall survival [Table 1]. Interestingly, the levels of TCTP were higher in metastatic lesions than in the corresponding primary gallbladder (GBC)^[22]^ and colorectal carcinoma (CRC)^[27]^.

**Table 1:** TCTP expression and clinical outcomes

**Cancer**	**Expression level**	**Histological and prognostic features**	**Refs**
Breast	High	Poor differentiation Short survival	[18] [19]
Epithelial ovarian cancer	High	Poor differentiation Short survival Lymph node metastasis	[20]
Cervical cancer	High	Poor differentiation Lymph node metastasis	[21]
Gallbladder cancer	High	Poor differentiation Metastasis Short survival	[22]
Pancreatic ductal adenocarcinoma	High	Lymph node metastasis Poor differentiation	[23]
Glioma	High	Poor differentiation Short survival	[24] [25]
Colorectal cancer	High	Poor differentiation Metastatic Short survival	[26] [27]
Prostate	High	Poor differentiation Metastasis Short survival	[28] [29]
Lung adenocarcinoma	High	Poor differentiation Short survival	[30]
NSCLC	High	Tumor size Short survival	[31]
Cholangiocarcinoma	High	Short survival	[32]
Neurofibromatosis type 1	High	Malignant phenotype	[33]
Neuroblastoma	High	Poor differentiation Short survival	[34]

TCTP: Translationally controlled tumor protein.

In breast cancer tissues, TCTP expression levels were higher compared to the corresponding normal tissues, and, notably, a high TCTP status was positively correlated with the pathological grade and was associated with shorter overall survival^[18]^. Interestingly, TCTP was enriched in PKH26 dye-retaining human normal mammary stem cells^[35]^. Further, TCTP enrichment was recorded in poorly differentiated high-grade BC tumors^[18]^, the latter known to contain more cancer stem cells (CSCs) than lower-grade tumors^[35]^. Relevant to this, a high TCTP status has also been correlated to the presence of mutated P53 tumors and with high proliferative activities^[18]^. Loss of p53 function enabled acquisition of stem cell properties and led to Myc activation, thereby increasing the expression of a mitotic signature identifying BC patients at high risk of mortality and relapse^[36]^.

Moreover, TCTP mRNA levels were significantly upregulated in ovarian cancer (OC) organoids generated from pluripotent stem cells (iPSC) of patients bearing the germline pathogenic breast cancer susceptibility gene 1 (*BRCA1*) mutation^[37]^. These findings highlight the potential role of TCTP as a biomarker in OC since patient-derived-organoids (PDOs) are a clinically valuable representation of the sourcing tumor^[38]^. This again echoes the above-mentioned data showing that TCTP levels are higher in poorly differentiated tissues compared to the well-differentiated ones in several tumors. Understanding the relationships between the BRCA1 genomic status and the higher level of TCTP in prognostic terms, in OC and additional tumors, may represent an important future investigation.

### TCTP as a predictive marker

We have shown that TCTP, in the phosphorylated form, was a clinically relevant biomarker for a more aggressive BC^[19]^. TCTP was specifically phosphorylated by Polo-like kinase 1 (PLK1) on Ser46 and Ser64 residues^[39-41]^, both located in the flexible loop of the protein, as reported by Malard *et al.*^[42]^. PLK1 was a crucial player in mitotic progression^[43]^. It was highly expressed in preinvasive in situ breast carcinomas^[44]^, and correlated with high Ki-67 levels, TP53 mutations and poor clinical outcomes in primary BC^[45,46]^. Further, overexpression of PLK1 played a critical role in tumors that have escaped estrogen deprivation therapy^[46,47]^, and was a strong predictor of worse survival in a large cohort of ER-positive BC patients^[46-48]^.

**Figure 3:**
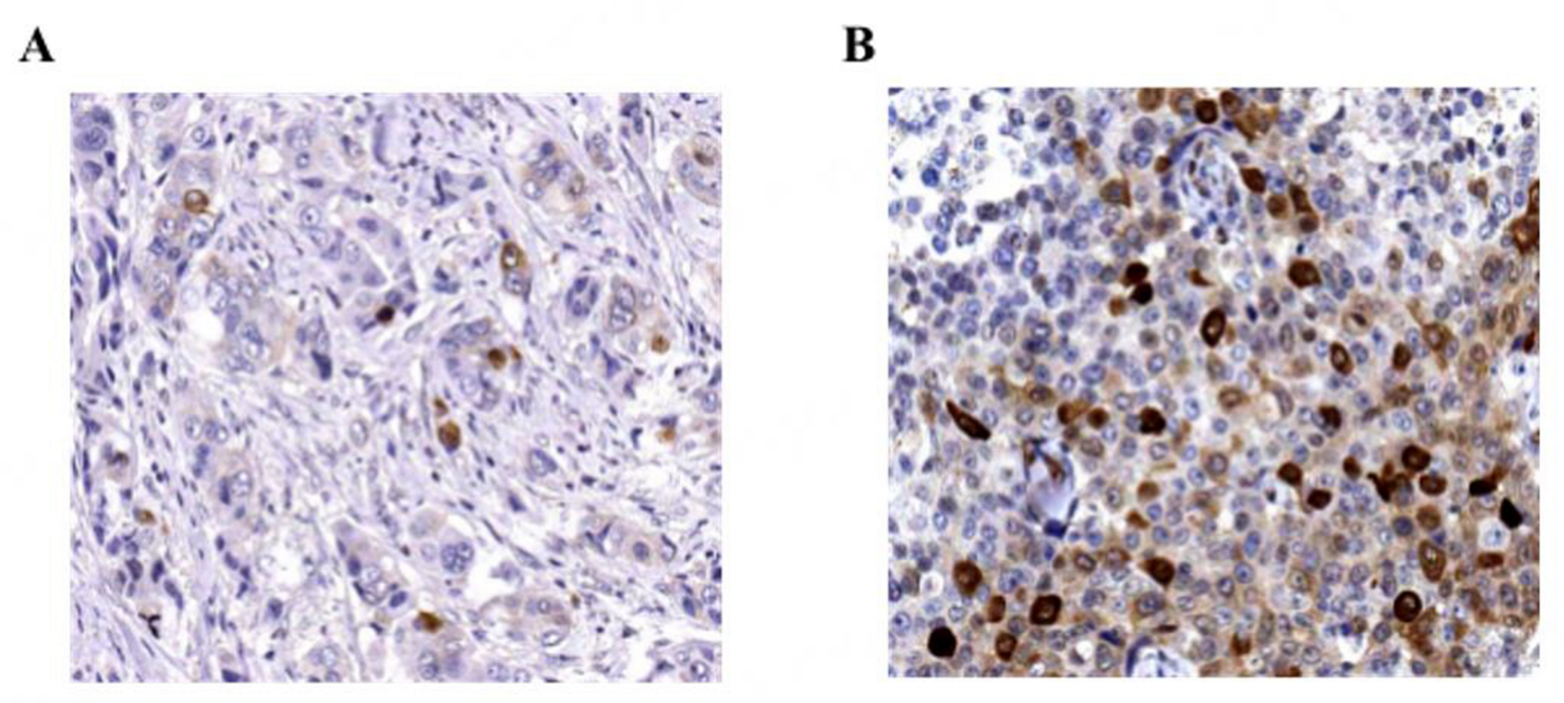
Correlation between Phospho-TCTP expression and response to trastuzumab therapy. Representative images of the immunohistochemical staining of phospho-TCTP in HER2 + BC patients (A) responsive and (B) non-responsive to trastuzumab. From Lucibello *et al.* “Phospho-TCTP as a therapeutic target of dihydroartemisinin for aggressive breast cancer cells”. Oncotarget, 2015 [Figure 6]^[19]^. BC: Breast cancers; HER2: human epidermal growth factor receptor-2; TCTP: translationally controlled tumor protein.

We and others have shown that inhibition of PLK1 impaired TCTP phosphorylation^[19,39,40]^, suggesting that a functional PLK1/TCTP axis could be critical for cancer progression. Consistently, both high levels of phospho-TCTP and PLK1 were found in neuroblastoma from patients with adverse prognostic factors^[34]^, in agreement with our data showing that high levels of phospho-TCTP were correlated with high histological grade and with worse pathological parameters in primary BC tissues. Interestingly, the number of phospho-TCTP positive cells significantly increased (> 10%) when tumors were resistant to trastuzumab therapy (first line of treatment) and progressed towards the metastatic stage [Figure 3]^[19]^. These findings suggest that phospho-TCTP may be a promising independent prognostic biomarker and a possible predictor of response to treatment. Tumors endowed with a higher fraction of cancer cells with a positive phospho-TCTP staining may exhibit increased drug resistance. A high phospho-TCTP status may allow classification of patients into responders and non-responders for trastuzumab therapy. Thus, targeting these cells with dihydroartemisinin (DHA) (see below) may improve long-term clinical outcomes, as recently suggested by our findings in HER2+BC cells resistant to trastuzumab therapy^[49]^.

### TCTP as a non-invasive diagnostic biomarker

Accessible biomarkers are a valuable and promising non-invasive tool for early diagnosis of BC. Serum tumor biomarkers such as carcinoembryonic antigen (CEA), cancer antigen (CA)-15-3, and CA-125 have been used for early detection of metastatic breast cancer (MBC). However, expression levels of these markers may be affected by anti-hypertensive medications or inflammatory diseases^[50]^, thus raising concerns about their diagnostic accuracy.

TCTP has been shown to be secreted during allergic reactions and to promote immunoglobulin E (IgE)-mediated activation of mast cells and basophils. Its levels also increased in the serum of patients affected by rheumatoid arthritis (RA), suggesting a potential role as a biomarker in autoimmune and inflammatory disease^[51,52]^.

Recently, a transcriptome profiling analysis has shown that TCTP mRNA levels increased in the saliva of BC patients compared to healthy ones^[53]^. In addition, ischemia and hypoxia induced TCTP secretion in blood samples of CRC patients, suggesting that circulating TCTP levels may play a role in CRC progression^[27]^. Moreover, the levels of TCTP were higher in the plasma of patients with cervical cancer^[21]^ or squamous cell carcinomas (SCCs)^[54]^ when compared to healthy subjects, suggesting its potential role as a novel non-invasive biomarker in several cancer types.

Extracellular vesicles (EVs) have also been proposed as reliable diagnostics biomarkers for the early detection of cancer. EVs are a heterogeneous population of vesicles that comprise three main classes: exosomes, shed microvesicles, and apoptotic bodies. EVs play a role in regulating intercellular communications by transferring biological information like proteins, lipids, and nucleic acids between cells^[55]^.

TCTP is a leaderless protein that could be secreted through the non-classical exosome pathway by cancer cell lines^[56]^. Recently, it has been shown that TPT1 transcripts were highly expressed and enriched in all EV subtypes from the human colon cancer LIM1863 cell line, thus speculating that it could be translated upon EV uptake in recipient cells^[57]^. It has been shown that the TCTP protein was sequestered into exosomes and released from endothelial cells in patients with pulmonary arterial hypertension (PAH). TCTP uptake from the circulation by pulmonary artery smooth muscle cells induced aberrant vascular remodeling, suggesting TCTP is a relevant biomarker in PAH disease^[58]^.

Altogether, all these data suggest the potential of TCTP as a non-invasive biomarker in several diseases and pave the way for further studies strengthening its potential extracellular role in cancer diagnosis and prognosis.

### TCTP as an immune-resistance factor

Oxidative stresses, lack of nutrients, radio and chemotherapy can trigger cell death responses. While programmed cell death ensures, at least initially, the removal of apoptotic bodies by phagocytes, death by necrosis results in the release of protein factors, called damage-associated molecular patterns (DAMPs), in the extracellular environment. These proteins have a well-defined intracellular function. However, when they are released in the tumor microenvironment (TME) through interaction with specific receptor molecules, such as Toll-like receptors (TLRs) or receptors for advanced glycation products (RAGE) present on epithelial cells or resident inflammatory cells, they induce recruitment of immune-inflammatory cells. Among them, there are immune cells with potent immunosuppressive activities as the myeloid-derived suppressor cells (MDSCs)^[59,60]^.

Recently, it has been shown that TCTP was a crucial immunosuppressive danger factor released by dying tumor cells in the TME. TCTP promoted the recruitment of polymorphonuclear MDSCs (PMN-MDSC) into TME, which in turn blocked the antitumor function of CD8+T cells and NK cells. In detail, TCTP, through its binding to TLR2 on myeloid cells, stimulated the CXCL1 family chemokine expression, which in turn recruited PMN-MDSCs. This specific pathway was sufficient for the recruitment of PMN-MDSCs as no additional signals, such as G-CSF and GM-CSF, were required^[61]^. Notably, in this setting, the inhibition of TCTP, with a specific monoclonal antibody (55F3) or with DHA (see below), decreased MDSCs in the TME, inhibited tumor growth, thereby enhancing the efficacy of the immune checkpoint blockade (ICB)^[61]^. Thus, TCTP is a relevant player in such immunosuppressive circuits, and its inhibition may be effective in clinically viable combinatorial settings. Consistently, interrogating a TCGA colorectal data set showed that TCTP expression levels were negatively correlated with an antitumor immune signature of cytotoxic lymphocyte or NK cells. In CRC, a higher TCTP expression was elevated specifically in tumor cells and correlated with advanced disease and, notably, with PMN-MDSCs (CD15+ cells) representation within the tumor^[61]^. This latter observation correlates with the fact that the mesenchymal transition of high-grade breast carcinoma may be induced by MDSCs infiltrating the primary tumor^[62]^.

The programmed death ligand-1 (PD-L1) is an immune checkpoint molecule and informative biomarker for anti-PD-1 therapy. Aberrant expression of PD-L1 may be induced through the dysregulation of several oncogenic pathways and contributes to immune escape^[63,64]^. TCTP was recently shown to induce resistance to anti-PD-L1 therapy, decrease T cell trafficking to the tumor and confer resistance to cytotoxic T lymphocyte-mediated tumor cell killing. In this frame, TCTP induced activation of EGFR-AKT signaling, and notably, the phosphorylation of TCTP by PLK1 was required for EGFR-AKT signaling and for establishing an immune-resistant phenotype^[65]^. It follows that inhibition of TCTP by DHA enhanced the efficacy of T cell-mediated therapy. Consistently, the expression of the TCTP mRNA was significantly higher in the patients unresponsive to anti-PD-L1 therapy, and it inversely correlated with T cell infiltration and CD8+T cell signatures in various types of cancer^[65]^.

All these findings raise the intriguing possibility that TCTP might mark a subpopulation of cancer cells bearing immune-evasive properties. Finally, TCTP levels and status may predict the efficacy of anti-PD-1/PD-L1 immunotherapy, and, again, TCTP itself could be an actionable target for combinatorial therapies improving the response to T cell therapy or immune checkpoint blockade.

## TCTP MAY MARK POORLY DIFFERENTIATED CELLS WITH STEM-LIKE PROPERTIES AND RESISTANCE TO THERAPY-INDUCED STRESS

Epithelial-mesenchymal transition (EMT) is a property of epithelial stem cells^[66]^. EMT is involved in tissue remodeling during embryonic development and in several pathophysiological processes in adult life, such as invasion and spread of tumor metastasis. During EMT, cells gradually acquire a mesenchymal cell phenotype characterized by a loss of contact with neighboring cells by changing cell shape and increasing the ability of migration and invasion. EMT is the first step towards metastasis. Disseminated cancer cells then undergo a mesenchymal-epithelial transition (MET) to re-initiate tumor growth. Epithelial and mesenchymal states are not endpoints of a transition but rather the expression of reversible phenotypic states. This phenotypic plasticity enables cancer cells to adapt to specific microenvironments, metabolic, immune, and therapeutic challenges^[67-69]^. Reactivation of developmental programs may be a critical step in the progression of cancers. Thus, genes and signaling pathways playing key roles in embryonic development pathways are often reactivated or dysregulated during tumorigenesis and metastasis^[70]^.

TCTP knockout mice and TCTP-deficient mutants of Drosophila died in the early stage of embryogenesis^[71,72]^, suggesting defects in embryo development. A recent study by Kwon YV and colleagues has shown that TCTP was required for maintaining Drosophila intestinal stem cells (ISCs) during normal homeostasis and tissue damage. In such a setting, TCTP increased Akt1 levels and its phosphorylation, which in turn promoted stem cell proliferation^[73]^. TCTP was enriched in PKH26 dye-retaining breast cancer stem cells, as previously mentioned^[18]^, and in glioma stem cells^[74]^.

TCTP has been shown to be a positive regulator of EMT. TCTP overexpression in polarized epithelial LLC-PK1 cells enhanced their cell motility, and invasiveness via mTORC2/Akt/GSK3b/b catenin pathway and promoted EMT-related markers and morphological changes^[75]^. TCTP was a target of transforming growth factor-β1 (TGF-β1), a potent inducer of EMT, in lung carcinoma cells. TCTP overexpression induced the protease urokinase plasminogen activator (uPA), which in turn induced extracellular matrix degradation, up-regulation of mesenchymal markers like vimentin, and reduction in epithelial markers, such as E-cadherin^[76]^.

Several studies have shown a strong correlation between the expression levels of TCTP and the degree of metastasis [Table 1]. Importantly, silencing of TCTP suppressed pulmonary metastasis in melanoma-bearing mice^[75]^, or liver metastasis in an *in vivo* model of GBC metastasis^[22]^. These data highlight the role of TCTP in the progression of the disease and its potential as a target in metastatic lesions.

Reorganization of cytoskeleton and changes in cell shape are essential for cancer cell mobility^[67]^. In Xenopus XL2 cells, TCTP was localized in a subset of actin-rich fibers of migrating cells, therefore, may regulate cell shape. Indeed, its reduction in XL2 and HeLa cells provoked drastic MT-dependent shape change^[77]^. Along this line, in LLC-PK1-renal proximal tubular epithelial cells, overexpression of TCTP induced a rearrangement of actin cytoskeleton and formation of stress fibers, which played a crucial role in cell mobility regulation^[75]^.

We have observed that depletion of TCTP in breast cancer cells induced alterations in cell morphology, specifically an enlarged size and flattened shapes^[78]^. Notably, in several cancer cells, a decrease in TCTP levels significantly reduced cell migration and invasion^[22,31,75]^. We have also shown that a reduction of TCTP in its phosphorylated form induced an increase in microtubule density.

Thus, the phosphorylated and non-phosphorylated forms of TCTP must be kept in equilibrium to maintain the dynamic instability of microtubules and the cell shape. We also found that phospho-TCTP activity was crucial during mitosis, reminiscent of its mentioned link to cell proliferation^[49]^.

We have also observed that TCTP is a critical survival factor that protects cancer cells from oxidative and metabolic stresses. Notably, arsenic trioxide (ATO), a pro-oxidant agent, induced up-regulation of TCTP, suggesting that chemotherapeutic treatments, through induction of TCTP expression, may select cells with a survival advantage^[78]^. This is intriguing since adaptation to stress and invasive ability are recognized features of stem-like cancer cell subpopulations. In this context, it is worth noting that EMT was shown to be an important propeller of cancer stem cell emergence^[66]^.

Consistent with these data, in several tumors such as breast, lung, CRC and melanoma, a high TCTP status increased resistance to radio- and/or chemotherapy^[79-82]^.

In chemo-resistant cells, the induction of TCTP was regulated through phosphoinositide-3-kinase (PI3K)/Akt/mTORC1 pathways^[79]^, whose aberrant activation during malignant progression might result in loss of control of cell growth and survival, and the development of drug resistance^[83]^.

Radiation may induce DNA damage by generating reactive oxygen species (ROS). TCTP knockdown sensitized cancer cells to radiation-induced DNA damage, reminiscent of its cytoprotective role under oxidative stress conditions^[78]^, and activated p53, which in turn prevented proliferation of damaged cells^[81]^, consistent with data showing that p53 and TCTP work as reciprocal regulators^[18]^.

High levels of TCTP correlated with resistance to radiotherapy in high-grade glioma patients^[25]^. High-grade glioma tumors are characterized by an enrichment of CD133-positive cells with stemness properties, which may persist after therapy leading to tumor relapse^[84]^. In this context, TCTP could play a crucial role, as it has been shown that it is essential for cell proliferation and survival of primary glioma CD133-positive cells^[74]^.

Altogether, these findings suggest that high TCTP levels could delineate a population of stem-like cancer cells with metastatic potential and pronounced stress adaptive properties, including chemo- and radio-resistance. This fits well with the above observations that TCTP could be a relevant indicator of high-grade malignancy.

### Genomic analysis of TCTP in BC tumor

**Figure 4:**
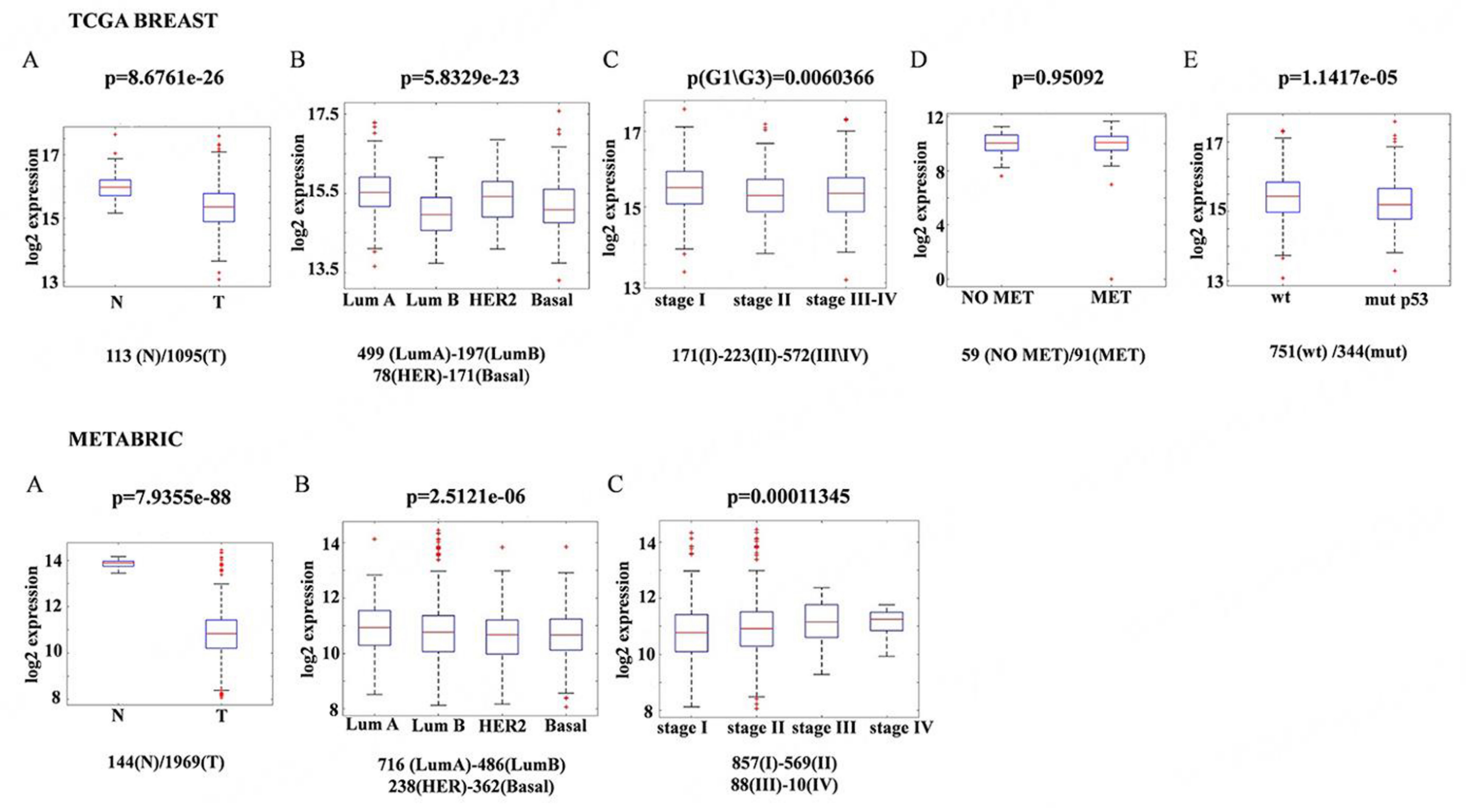
TPT1/TCTP transcripts in BC and normal tissues. Boxplot from TPT1/TCTP RNAseq gene expression data of TCGA (upper panel) and METABRIC databases (Lower panel). (A) in normal tissue and primary breast cancer tissue; (B) in different PAM50 breast cancer subtypes; (C) in different stages of the tumor; (D) in metastatic (91 samples) *vs.* no metastatic tumors (59 samples); (E) in TP53 mutated tumors *vs.* TP53 wild-type tumors. BC: Breast cancers; MET: mesenchymal-epithelial transition; METABRIC: molecular taxonomy of breast cancer international consortium; TCGA: the cancer genome atlas; TCTP: translationally controlled tumor protein; TPT1: tumor protein, translationally-controlled 1.

The Cancer Genome Atlas Breast Invasive Carcinoma (TCGA-BRCA) or Molecular Taxonomy of Breast Cancer International Consortium (METABRIC) databases showed a higher expression of TPT1/TCTP in normal breast tissues than in primary breast tumors. In addition, these analyses showed no differences in mRNA levels: (i) among the PAM50 subtypes of BC; (ii) among specific stages of the tumor; (iii) in metastatic *vs.* no metastatic tumors; (iv) in TP53 mutated tumors *vs.* TP53 wild-type tumors [Figure 4].

**Figure 5:**
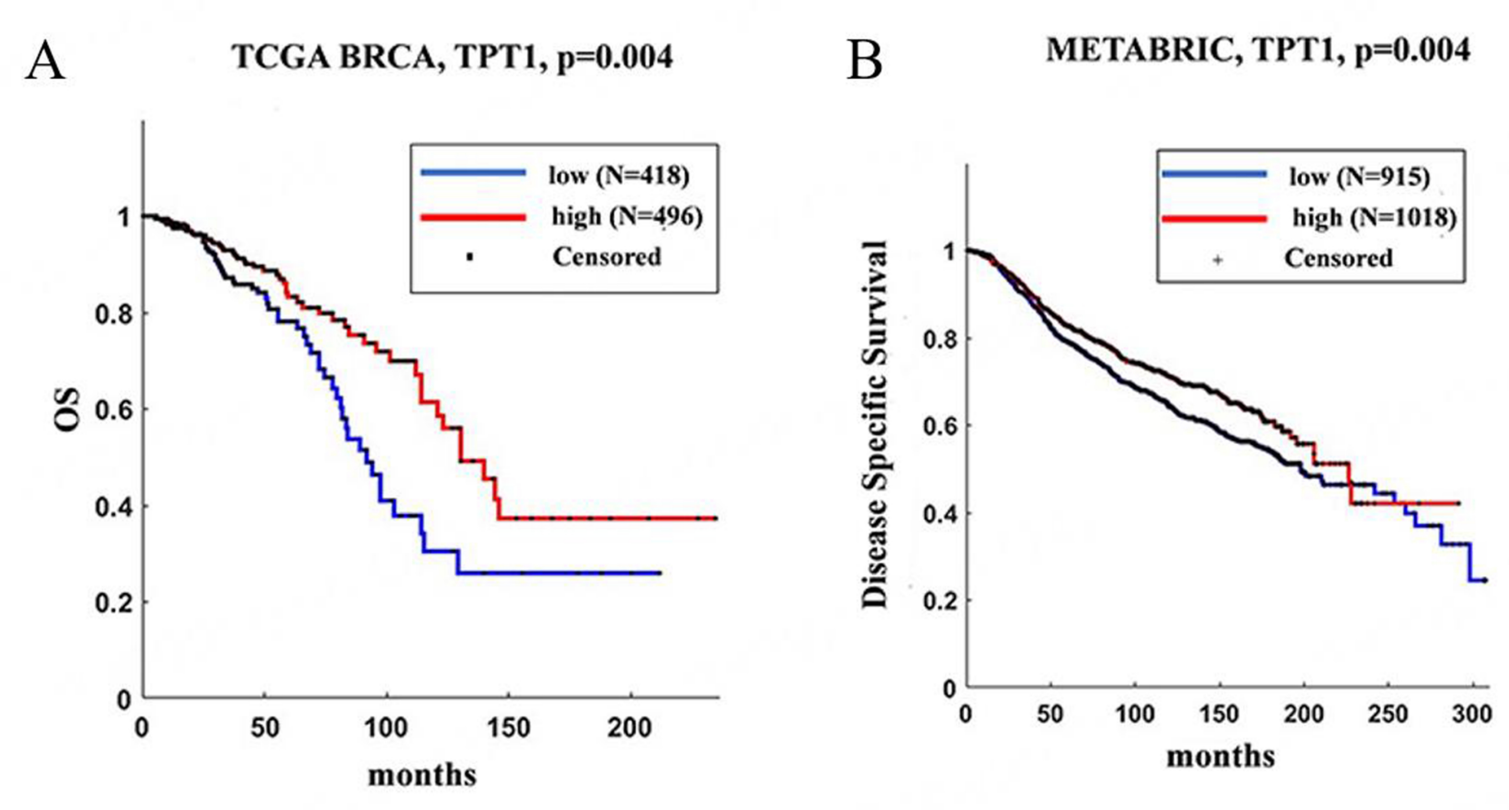
High TCTP levels correlate with better clinical outcomes in BC patients. (A) TCGA survival curve (B) and Disease-Specific Survival in METABRIC dataset of BC patients separated according to TPT1 expression levels. BC: Breast cancers; BRCA: breast cancer susceptibility gene; METABRIC: molecular taxonomy of breast cancer international consortium; TCGA: the cancer genome atlas; TCTP: translationally controlled tumor protein; TPT1: tumor protein, translationally-controlled 1.

Moreover, the survival analysis from the Kaplan-Meier plot showed a positive correlation between high TPT1/TCTP mRNA levels and patient survival [Figure 5]. All these data are in apparent disagreement with the aforementioned data and suggest that mRNA levels of TCTP do not predict protein levels^[6]^.

**Figure 6:**
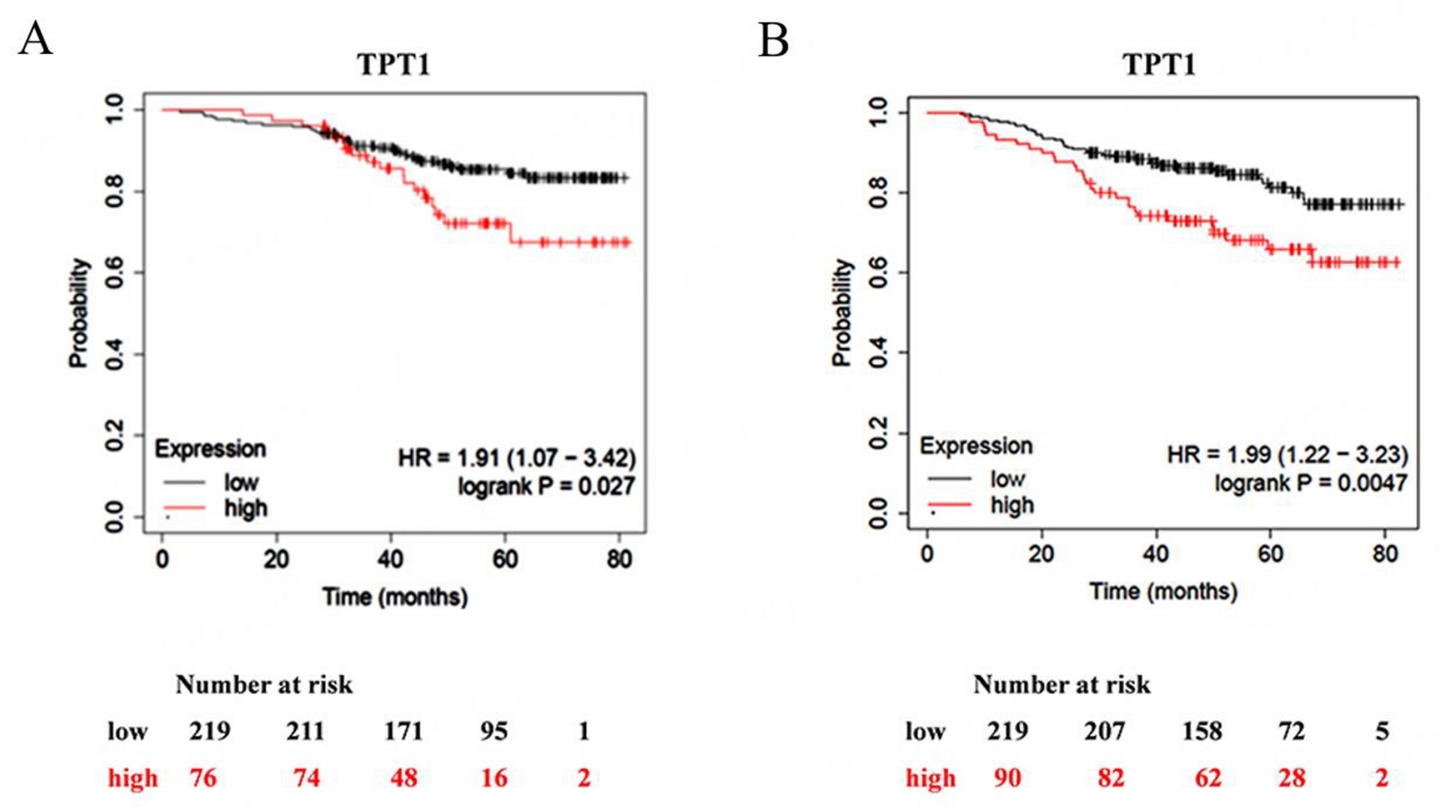
Overall survival is shorter in BC patients with higher TCTP expression. Kaplan-Meier analysis of overall survival in BC patients based on KM plotter database of: (A) PAM50 HER2 and (B) PAM50 basal subtypes. BC: Breast cancers; HER2: human epidermal growth factor receptor-2; TCTP: translationally controlled tumor protein; TPT1: tumor protein, translationally-controlled 1.

Things change when considering specific features of BC patients: when the analysis was restricted to estrogen receptor (ER)-negative tumors, characterized by more aggressive behavior [Figure 6], a high TCTP status positively correlated with shorter overall survival, thus suggesting the high degree of heterogeneity among BC subtypes.

**Figure 7:**
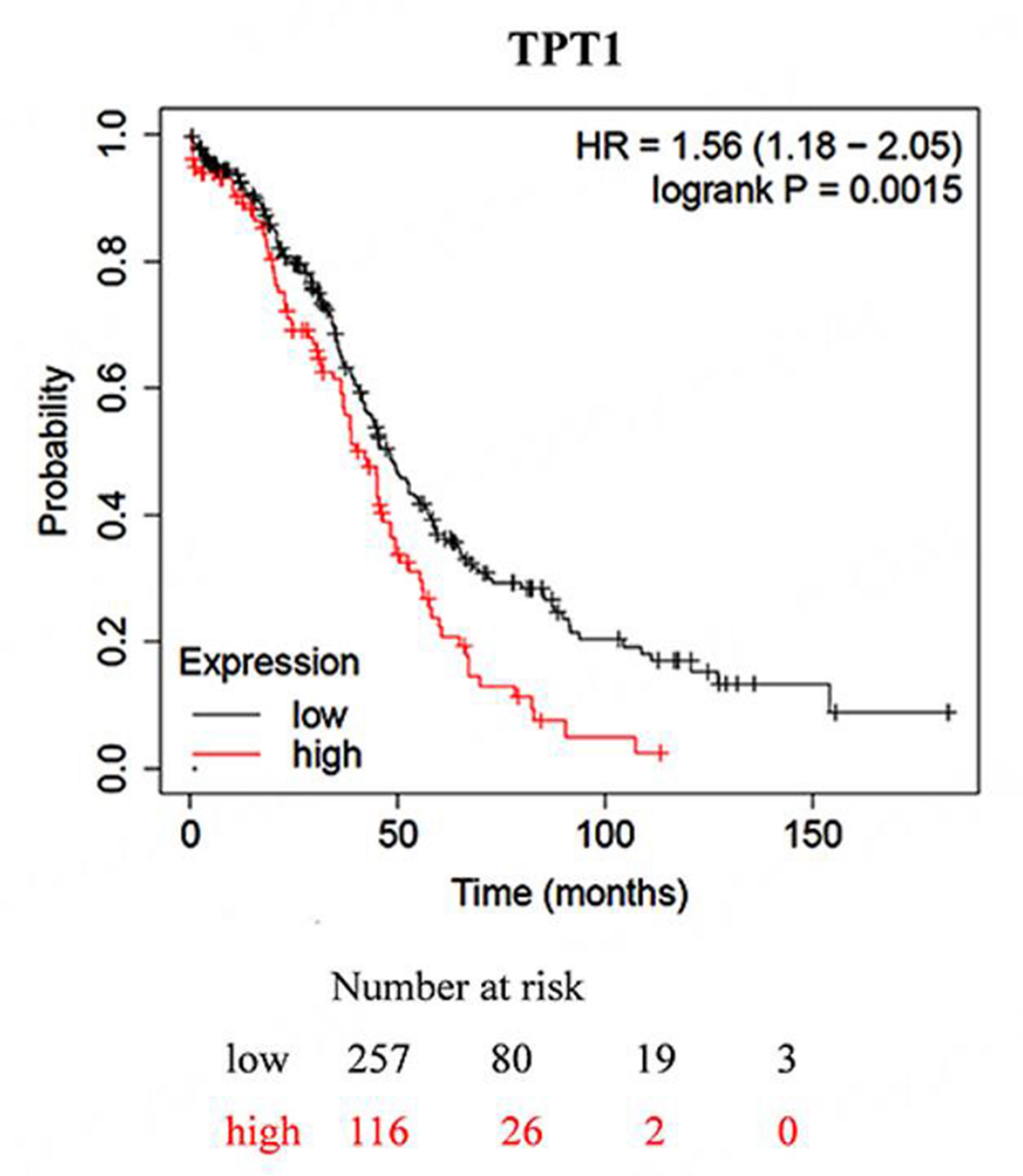
Overall survival is shorter in OC patients with higher TCTP expression. Kaplan-Meier analysis of overall survival in OC patients based on KM plotter dataset. OC: Ovarian cancer; TCTP: translationally controlled tumor protein; TPT1: tumor protein, translationally-controlled 1.

In OC, instead, high levels of TCTP mRNA and protein were both significantly associated with poor overall survival [Figure 7]. Since OC is characterized by a history of sequential relapses after a second-line therapy^[85]^, and given the involvement of OC cancer stem cells (CSC) in OC relapse^[86]^, this indirectly links TCTP to the CSC biology, thus echoing the previous observation. See the availability of data and material for more details.

## STRATEGIES TARGETING TCTP

For a more systematic view of the potential therapeutic approaches toward TCTP, we will divide the following discussion into subparagraphs.

### Non-coding RNA

Non-coding RNA (ncRNAs) play key regulatory roles in oncogenesis through epigenetic, transcriptional, post-transcriptional, and translation modulation. ncRNAs can be used as biomarkers or can be therapeutically targeted in cancer therapy^[87]^. They are grouped into two main types according to transcript size: small (< 200 nucleotides; ncRNAs) and long (> 200 nucleotides; lncRNAs)^[88]^.

Post-transcriptional gene regulation of TPT1 by potential ncRNAs in breast cancer remains largely unexplored. Recent works have shown that the lncRNA TPT1 antisense RNA 1 (TPT1-AS1) was dysregulated in tumors and associated with patient prognosis and clinically relevant features^[89]^. Conversely to TCTP, TPT1-AS1 was downregulated, and this predicted poor prognosis in BC. TPT1-AS1 played a role as a tumor suppressor gene^[90,91]^, and notably, it was subjected to epigenetic regulation through DNA methylation. Elango and colleagues have shown that TPT1-AS1 was the most induced lncRNA in response to DNA methyltransferase inhibition in triple-negative breast cancer (TNBC) models^[92]^. DNA methyl transferase inhibitors reverse epigenetic alterations, resulting in reactivation of tumor suppressor genes, which in turn leads to cell cycle arrest and/or apoptosis, and thus suggests that re-expression of TPT1-AS1 could be an approach to be pursued in breast cancer therapy.

More in detail, the TPT1-AS1 is a transcript from the antisense strand of *TPT1* gene that may positively regulate the expression of TPT1. Consistent with this, TPT1-AS1 promoted tumor progression by upregulating TPT1 levels in epithelial ovarian cancer (EOC). In addition, the high expression of TPT1-AS1 was associated with unfavorable clinic-pathological features and poor prognosis in EOC^[93]^. The dual functions of TPT1-AS1 suggest that its role should be carefully considered in a tissue-specific manner. Further studies are therefore required to characterize the complex interaction network that regulates TPT1-AS1 activity in a specific tissue or context and to understand its underlying mechanisms.

### Clinically available compounds

Drug repositioning is a promising strategy to identify new therapeutic applications of drugs already approved by regulatory agencies for other diseases. The use of clinically approved or advanced phase compounds is justified by the need to maximize the timing for therapeutic advancements since those compounds already have a consolidated safety profile and a characterized pharmacokinetic/pharmacodynamic profile. Thus, those compounds are in principle amenable to phase 2 studies. Below we will list some of the compounds falling into this category, whose mechanism of action is directly or indirectly linked to perturbing the levels or post-translational status of TCTP [Tables 2-4].

**Table 2:** DHA as a repurposed drug targeting TCTP

**Effect**	**Studies**
Inhibition BC cell growth. Chemosensitization	Lucibello *et al.* 2015^[19]^
Inhibition of GBC migration and invasion. Inhibition of GBC metastasis. Improved survival in mice	Zhang *et al*. 2017^[22]^
Enhancement of anti- HER2 antibody therapies	D’Amico *et al*. 2020^[49]^
Inhibition of tumor growth Reduction of MDSCs in TME	Hangai *et al*. 2021^[61]^
Enhancement of the efficacy of T cell therapy	Lee *et al*. 2022^[65]^

BC: Breast cancers; DHA: dihydroartemisinin; GBC: gallbladder; HER2: human epidermal growth factor receptor-2; MDSCs: myeloid-derived suppressor cells; TCTP: translationally controlled tumor protein; TME: tumor microenvironment.

**Table 3:** Sertraline and thioridazine as repurposed drugs targeting TCTP

**Effect**	**Studies**
Inhibition cell growth and induction of apoptosis. Reduction of mammosphere-forming efficiency	Amson *et al*. 2012^[18]^ Tuynder *et al*. 2004^[105]^
Inhibition of migration and invasion Inhibition of tumor growth	Boia-Ferreira *et al*. 2017^[106]^
Reduction of the number of prostate cancer stem cells	Chinnapaka *et al*. 2020^[107]^
Enhancement of the efficacy of DNA-damaging therapy and PARP1 inhibitor	Li *et al*. 2017^[108]^

TCTP: Translationally controlled tumor protein.

**Table 4:** Rapamycin as a repurposed drug targeting TCTP

**Effect**	**Studies**
Inhibition of MPNSTs cell growth *in vivo*	Kobayashi *et al*. 2014^[33]^
Enhancement of the efficacy of cisplatin and doxorubicin	Bommer *et al.* 2017^[79]^
Inhibition of cell growth	Bommer *et al.* 2015^[113]^

MPNSTs: Malignant peripheral nerve sheath tumors; TCTP: translationally controlled tumor protein.

#### Dihydroartemisinin

**Figure 8:**
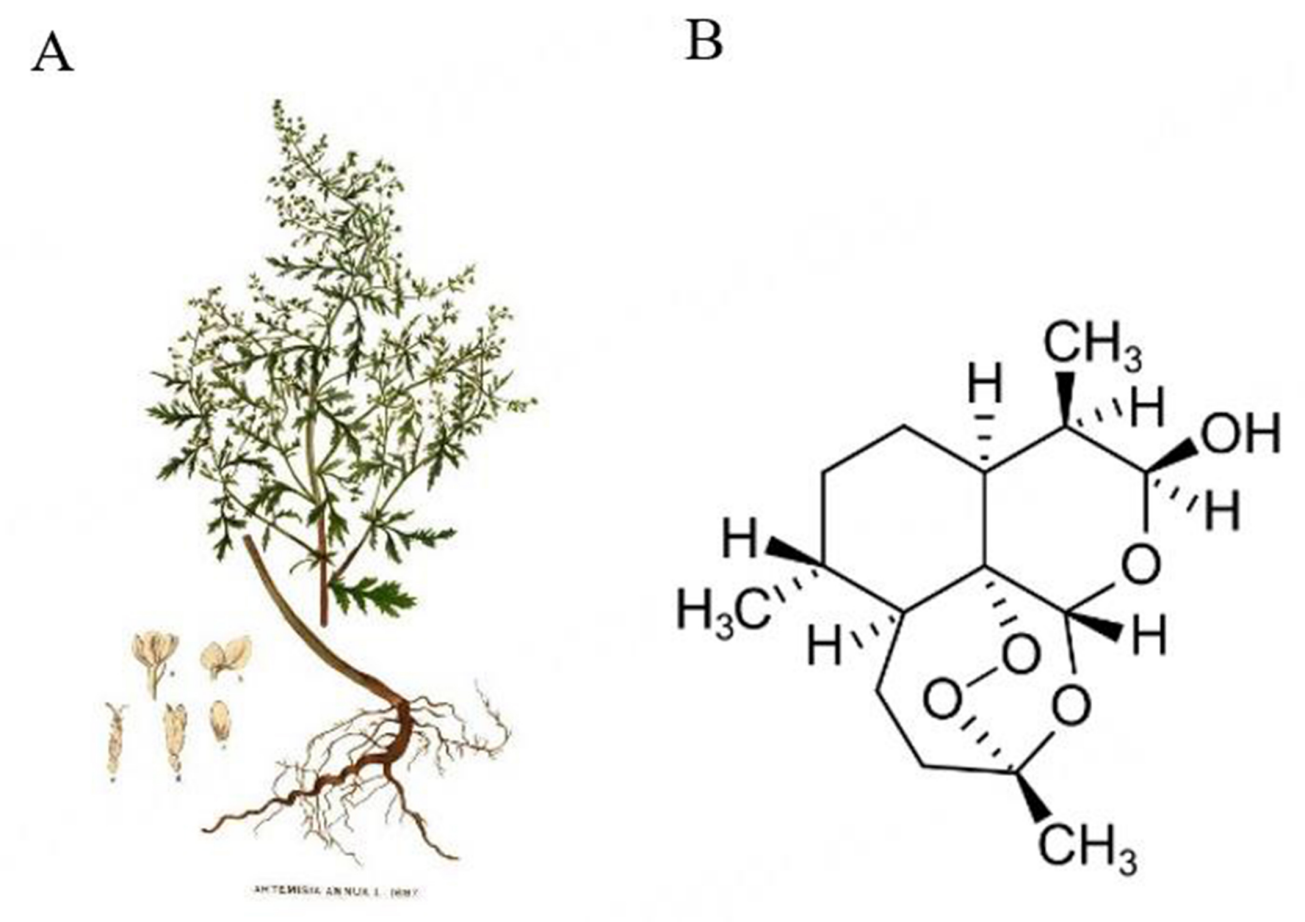
(A) Representative image of *Artemisia Annua*. https://commons.wikimedia.org/wiki/File:Artemisia_annua_-_001x.jpg. Created by: Oceancetaceen - Alice Chodura, Public domain, via Wikimedia; (B) Chemical structures of dihydroartemisinin. https://commons.wikimedia.org/wiki/File:Artenimol_(2).svg. Created by: Benff, CC BY-SA 4.0 <https://creativecommons.org.

One clinically available TCTP-targeting agent is DHA [Figure 8], the active metabolite of all artemisinin compounds (as artesunate, artemether). Artemisinin is the active principle in Artemisia annua. Artemisinin and its derivatives are a family of sesquiterpene trioxane lactone. These agents were discovered as anti-malarial agents by Dr. Youyou Tu, who received the Nobel Prize in Physiology or Medicine in 2015. Currently, the water-soluble derivative of artemisinin, Artesunate Amivas, has received approval for malarial patients from the Food and Drug Administration (FDA) and the European Medicine Agency (EMA).

Beyond the “anti-malarial effect”, these pharmacological compounds were selectively cytotoxic to cancer cells, as shown by numerous *in vitro* and *in vivo* studies^[94,95]^, which have led to the groundwork for the design of phase 1 clinical trials for solid tumors. These compounds have exhibited a good therapeutic index after long-term treatment in patients with metastatic breast cancer or cervix carcinoma^[96-98]^.

Several observations have shown that DHA is bound specifically to TCTP^[99-101]^. Further, DHA inhibited TCTP-dependent cell migration and invasion of GBC cells, and notably, DHA decreased GBC metastases and improved survival in tumor-bearing mice. TCTP is a clinically relevant target in GBC^[22]^; thereby, TCTP-targeting DHA could be an anti-metastatic strategy to be explored at an early stage of disease.

We have shown that the levels of phospho-TCTP were crucial for the mitotic process. By decreasing phospho-TCTP levels, DHA induced mitotic spindle aberrations and the formation of disorganized microtubule structures^[49]^. At least partially through this mechanism, DHA increased the sensitivity of BC cells to chemotherapy^[19]^ and to trastuzumab emtansine (T-DM1) in BC cells resistant to trastuzumab (first-line of treatment)^[49]^. T-DM1 is an anti-human epidermal growth factor receptor 2 (HER2) antibody-drug with a stable linker to emtansine (DM1), a microtubule inhibitor. Recently, it has been shown that T-DM1 reduced the risk of disease recurrence in patients with residual invasive BC after neoadjuvant therapy (NAT) comprised of HER2-targeted therapy and chemotherapy^[102]^. As partially suggested before, we may speculate that the addition of DHA to T-DM1 may allow a dose reduction for T-DM1 and could help prevent adverse side effects, therefore improving the quality of life for patients.

Targeting TCTP with DHA may offer an unforeseen rationale for combination treatments in cancer immunotherapy as well, according to what was previously mentioned^[61,65]^. Immunotherapy represents a promising therapeutic approach for TNBC, and recently, pembrolizumab (anti-PD1 antibody) plus chemotherapy was approved for the treatment of advanced TNBC with high PD-L1 expression^[103]^. However, resistance to immune-checkpoint inhibitors (ICIs), including aberrant activation of oncogenic signaling pathways and/or an immunosuppressive TME, is a significant challenge. Predictive factors, as well as discovering new actionable targets for combinatorial therapy, will improve the response rate of TNBC to ICIs. In this context, TCTP appears to be an interesting subject of investigation. Thus, targeting TCTP may enable chemosensitization of cancer cells previously subjected to conventional therapy and to biological agents [Table 2].

#### Sertraline and thioridazine

Sertraline (Zoloft) is a selective serotonin reuptake inhibitor. Thioridazine is an antipsychotic drug used to treat schizophrenia. These agents, used in the management of psychiatric disorders, have shown promising anticancer effects as well^[18,104]^.

Thioridazine and sertraline induced a significant reduction of TCTP levels, which in turn increased p53 levels, thus restoring the sensitivity of tumor cells to apoptosis. In addition, sertraline reduced the mammosphere-forming efficiency of ErbB2 cells, consistent with data showing that silencing of TCTP in ErbB2 cells resulted in a decreased ability to form mammospheres^[18,105]^. TCTP was also a critical target of sertraline and thioridazine in melanoma cells. Both drugs restored p53 function and inhibited invasion/migration and clonogenicity of melanoma cells by targeting TCTP. Notably, in vivo experiments on a mouse melanoma model have shown that sertraline was more effective in inhibiting tumor growth than dacarbazine, a chemotherapy agent that has been approved for treating advanced melanoma^[106]^. In prostate cancer cells, sertraline decreased TCTP, phospho-TCTP and survivin and induced apoptotic cell death. Further, sertraline inhibited the expression of cancer stem cell markers, CD44 and aldehyde dehydrogenase 1 (ALDH1), in prostate cancer cell cultures^[107]^. By using affinity purification-based proteomic profiling, Li *et al.* found that TCTP interacted with proteins involved in DNA repair. In breast cancer cells, inactivation of TCTP by sertraline enhanced UVC irradiation-induced apoptosis and increased the sensibility to etoposide and olaparib, a DNA-damaging drug and a PARP1 inhibitor, respectively^[108]^. Interestingly, such action may converge into the evoked chemosensitizing effect of TCTP [Table 3].

Whether sertraline and thioridazine may directly or indirectly perturb the function of TCTP is a matter of interesting ongoing debate^[109]^.

#### Rapamycin

Rapamycin is a natural anti-fungal antibiotic. Rapamycin and its analogs are currently being used in clinical as potent immunosuppressants and antiproliferative agents. Rapamycin inhibits the Target of Rapamycin Complex 1 (TORC1) activity with high specificity. TORC1 is a highly conserved protein kinase that belongs to the PI3K family. It is a crucial regulator of cell proliferation and growth that integrates and senses different signaling networks. Its signaling dysregulation is frequently observed in several pathological conditions and in aggressive and therapy-refractory cancers, among these BC^[83,110]^.

Studies in Drosophila melanogaster and structural studies in human models have shown the involvement of TCTP in TORC1 signaling. It has been reported that TCTP controlled cell growth and proliferation by positively regulating the Ras homolog enriched in brain (Rheb) activity, an important upstream activator of mTORC1^[14,72,111,112]^.

In addition, TCTP mRNA belongs to the class of 5’-TOP mRNAs (containing a 5’-terminal oligopyrimidine tract, 5’-TOP) whose translational activity is largely controlled through the PI3-K/Akt/mTORC1 pathway^[113]^. In colon cancer cells and in HeLa cells, serum stimulation increased the expression of TCTP, and this was inhibited by rapamycin or mTOR kinase inhibitors. In addition, in colon cancer, TCTP protein levels were upregulated by the mTORC1 pathway in response to 5-fluorouracile (5-FU) and oxaliplatin treatment, suggesting a protective role of TCTP against the cytotoxic action of those anticancer drugs. Consistent with this data, mTOR kinase inhibitors prevented the onset of this TCTP-driven drug resistance phenotype^[79]^.

Further, a positive feedback loop between TCTP and mTOR contributed to neurofibromatosis type 1 (NF1)-associated tumor, and rapamycin was effective in down-regulating TCTP expression, suggesting that the TCTP protein level was controlled by mTOR-dependent translational regulation^[33]^.

Recently, it has been shown that Rapamycin induced TCTP proteolysis, thereby enhancing the efficacy of DNA-damaging drugs, such as cisplatin and doxorubicin, in lung carcinoma cells, suggesting a novel strategy for enhancing chemosensitivity in lung cancers^[80]^.

Unfortunately, the mentioned compelling evidence has not yet generated clinically meaningful results, and this general observation is related to the poor activity of mTOR inhibitors observed so far in clinical trials. However, everolimus, an mTOR inhibitor, has been approved in combination with exemestane, an aromatase inhibitor, in HR^+^/HER2^-^ endocrine-resistant MBC^[114]^.

It is possible that multiple TCTP targeting agents may be combined to increase the effect: for example, preclinical studies have shown that the combination of artesunate, a specific inhibitor of TCTP (see above), and rapamycin was more effective in killing malignant peripheral nerve sheath tumors (MPNSTs) compared to the effect induced by exposure to a single drug^[33]^ [Table 4].

## CONCLUSION

**Figure 9:**
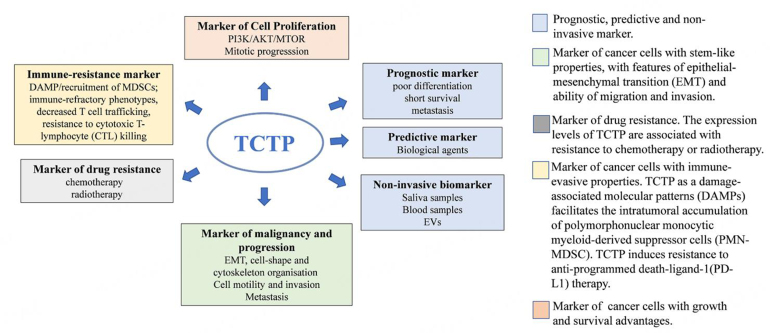
Schematic overview of the potential application of TCTP in cancer. EVs: Extracellular vehicles; MDSCs: myeloid-derived suppressor cells; TCTP: translationally controlled tumor protein.

TCTP may mark specific subpopulations of cancer cells with pronounced stress adaptation, stem-like and immune-evasive properties and capable of surviving conventional agents or targeted therapies [Figure 9].

**Figure 10:**
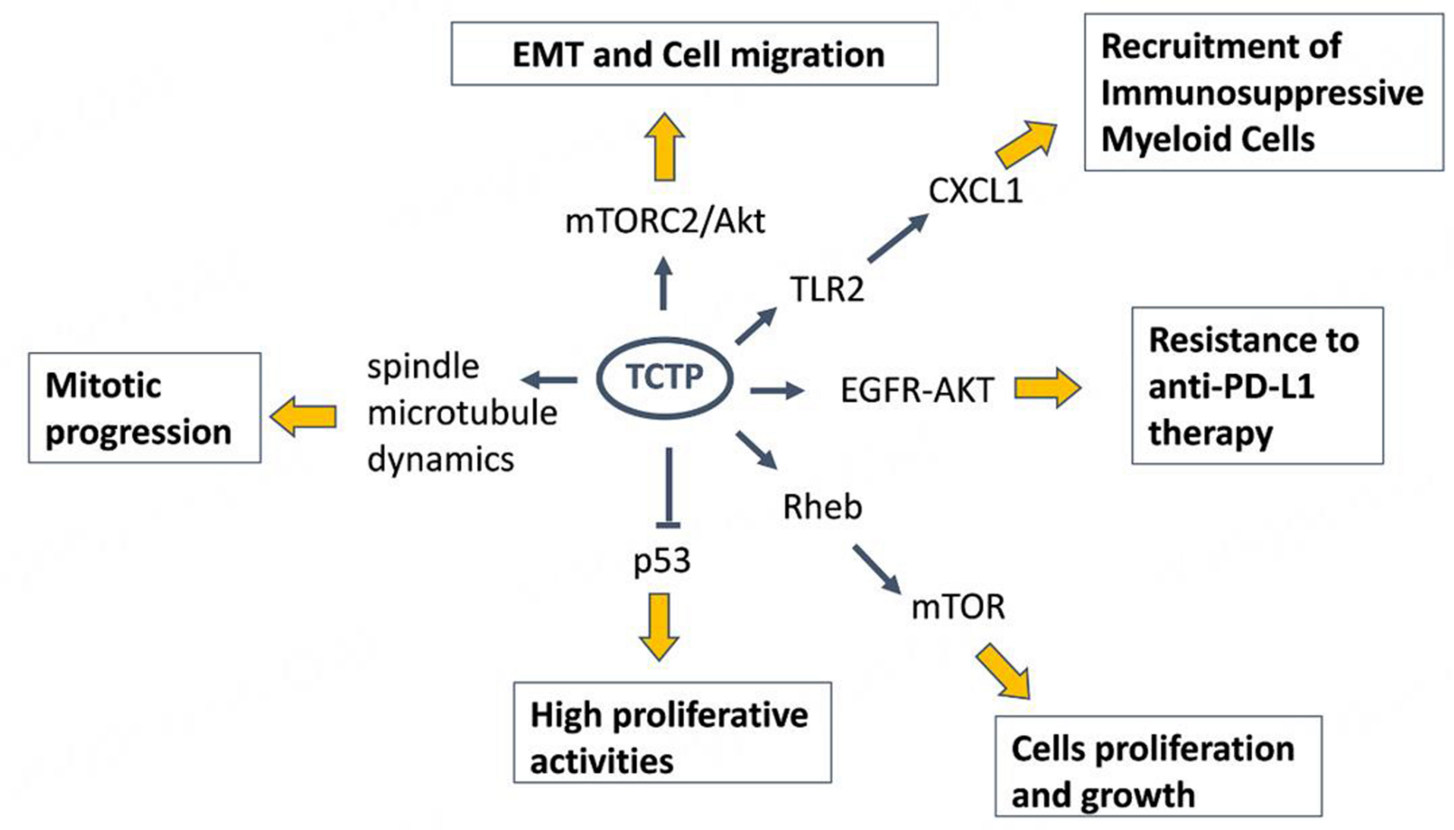
Pleiotropic functions of TCTP. A cartoon showing the major TCTP-related pathways. TCTP can promote cell proliferation by acting: (1) as a guanidine exchange factor for the GTP-binding protein Rheb, a crucial player in the mTORC1 pathway; (2) by inducing P53 degradation; (3) by regulating spindle morphology and mitosis progression, when it is phosphorylated by PLK1. TCTP can act as an immune-resistance factor via activation of TLR2 on myeloid cells, which in turn induces cytokines production and the recruitment of immune-suppressive cells. TCTP, in the phosphorylated form, can induce the activation of the EGFR/AKT pathway, thus promoting an immune resistance to anti-PD-L1 therapy. TCTP can promote cell migration and EMT markers through the mTORC2 pathways. EMT: Epithelial-mesenchymal transition; PD-L1: programmed death ligand-1; PLK1: polo-like kinase 1; TCTP: translationally controlled tumor protein.

It may work by interacting with key factors involved in cancer progression, including metabolic modulators. TCTP may also be involved in the tumor-TME crosstalk [Figure 10], and it may aid in stratifying specific cancer subsets. Thus, further studying TCTP and its complex behavior in cancer may reveal important knowledge towards patient stratification for both therapeutic and prognostic purposes. We also evoke the need to study the complex biology and the TCTP-modulating agents in more clinically relevant models, such as patient-derived-organoids. This may aid in solving some of the apparent contradictions in the behavior of this interesting and complex protein, down to a tumor- and patient-specific level. This effort is currently ongoing in our labs. Altogether, we suggest that TCTP may mark an aggressive sub-population of cancer cells that could emerge under the pressure of conventional therapies. Additionally, based on the preclinical evidence, we propose that TCTP could be an actionable target of clinically available compounds aimed at targeting TCTP highly expressing tumors, with the purpose of attenuating their resistance to therapy and progression. Targeting TCTP with DHA, sertraline or rapamycin holds promise for more effective synergistic combinations. This warrants further investigations, which are ongoing in our lab. Thus, we believe that two important factors may converge in delineating the study, and the targeting of TCTP can be an opportunity for personalized therapy. On one hand, the fact that TCTP marks a cell subpopulation of cancer cells, which represent obvious candidates for resistance to therapy, as also suggested by the prognostic value of both TCTP and phospho-TCTP levels; on the other hand, the availability of compounds with an established safety profile capable of reducing the levels of the protein and altering its phosphorylation state. What is mentioned here may represent a starting point for considering TCTP measurement and targeting as a translationally relevant opportunity.

### Statistics

Statistical analyses were performed using R software^[115]^. ANOVA or Student’s *t*-test was used for normally distributed data and for non-normally distributed data, as appropriate. *P*-values smaller than 0.05 were considered statistically significant.

## DECLARATIONS

### Availability of data and material

Data from the METABRIC study is deposited in the European Genome-phenome Archive and can be downloaded from European Genome-phenome Archive: METABRIC, (https://www.ebi.ac.uk/ega/studies/EGAS00000000083). The TCGA breast cancer dataset can be downloaded from the TCGA Data Portal website, (http://tcga-data.nci.nih.gov/tcga/).

### Copyright

© The Author(s) 2023.
